# Problem-Based mHealth Literacy Scale (PB-mHLS): Development and Validation

**DOI:** 10.2196/31459

**Published:** 2022-04-08

**Authors:** Lingmin Zhang, Pengxiang Li

**Affiliations:** 1 School of Journalism and Communication Guangzhou University Guangzhou China; 2 School of Media and Communication Shenzhen University Shenzhen China

**Keywords:** mobile health, mHealth literacy, instrument development, problem-based framework

## Abstract

**Background:**

Mobile devices have greatly facilitated the use of digital health resources, particularly during the COVID-19 pandemic. Mobile health (mHealth) has become a common and important way to monitor and improve health conditions for people from different social classes. The ability to utilize mHealth affects its effectiveness; therefore, the widespread application of mHealth technologies calls for an instrument that can accurately measure health literacy in the era of mobile media.

**Objective:**

We aimed to (1) identify the components of mHealth literacy for ordinary users and (2) develop a systematic scale for appropriately measuring individuals’ self-perceived mHealth literacy through a problem-based framework.

**Methods:**

We conducted an exploratory study involving in-depth interviews and observations (15 participants) in January 2020 and used exploratory factor analysis and confirmatory factor analysis to identify the components of mHealth literacy and develop an item pool. In February 2020, we conducted a pilot survey with 148 participants to explore the factor structures of items identified during the exploratory study. Subsequently, 2 surveys were administrated using quota sampling. The first survey (conducted in Guangdong, China) collected 552 responses during March 2020; we assessed composite reliability, convergent validity, and discriminant validity. The second survey (conducted in China nationwide) collected 433 responses during October 2021; we assessed criterion-related validity using structural equation modeling.

**Results:**

We identified 78 items during the exploratory study. The final scale—the Problem-Based mHealth Literacy Scale—consists of 33 items that reflect 8 domains of mHealth literacy. The first web-based survey suggested that mHealth literacy consists of 8 factors (ie, subscales), namely, mHealth desire, mobile phone operational skills, acquiring mHealth information, acquiring mHealth services, understanding of medical terms, mobile-based patient–doctor communication, evaluating mHealth information, and mHealth decision-making. These factors were found to be reliable (composite reliability >0.7), with good convergent validity (average variance extracted >0.5) and discriminant validity (square root of average variance extracted are greater than the correlation coefficients between factors). The findings also revealed that these 8 factors should be grouped under a second-order factor model (*χ*^2^/df=2.701; comparative fit index 0.921; root mean square error of approximation 0.056; target coefficient 0.831). The second survey revealed that mHealth use had a significant impact (*β*=0.43, *P*<.001) on mHealth literacy and that mHealth literacy had a significant impact (*β*=0.23, *P*<.001) on health prevention behavior.

**Conclusions:**

This study revealed the distinctiveness of mHealth literacy by placing mHealth needs, the ability to understand medical terms, and the skills in patient–doctor interactions in the foreground. The Problem-Based mHealth Literacy Scale is a useful instrument for comprehensively measuring individuals’ mHealth literacy and extends the concept of health literacy to the context of mobile communication.

## Introduction

### Background

Mobile technologies afford users ubiquitous access to information from the internet and to digital apps. Nearly half of the world’s current population (48.5%) owns a smart mobile phone, which has become the dominant form of access to the internet [1]. In countries such as China, this rate is even higher—99.6% of Chinese internet users rely on their mobile phones to access the internet [2]. The mobility, multimodality, and interactivity of mobile phones help individuals easily access digital health resources to manage and improve their health conditions. People can conveniently use health apps to monitor their health conditions, facilitate their physical exercises, acquire health information, and consult doctors. During the COVID-19 pandemic, mobile health (mHealth) has become particularly important. People rely on their mobile phones to check the status of the pandemic in their countries or neighborhoods, search for strategies for health prevention and protection, register for vaccines, and for many other activities. Accordingly, the adoption of mobile technologies for health-related practices has received widespread attention from researchers, practitioners, and policy makers [3,4]. As an extension of eHealth, mHealth generally refers to the use of mobile and wireless technologies, especially mobile phones and tablets, for health information and improving health care services and health outcomes [5].

Previous studies [6-8] have shown that digital health resources can benefit patients with chronic diseases by making self-monitoring more convenient, simplifying administrative procedures in health care, increasing access to health care services, reducing the cost of health care, and enriching resources in low- and middle-income countries. However, an individual’s health can be negatively impacted if they have poor mHealth skills or if they fail to adopt digital technology [9]. Biased digital health information, as well as the unskilled use of digital technology, can lead to misdiagnosis, which in turn may lead patients to try unapproved or unreliable therapies and to miss opportunities for optimal care [10,11]. Since mobile devices have become the main channel by which web-based health resources are accessed, a person’s mHealth literacy has become a factor in their health.

The concept of mHealth literacy builds upon that of eHealth literacy and is applied in the context of mobile and wireless technologies. eHealth literacy refers to “the ability to seek, find, understand, and appraise health information from electronic sources, and apply the knowledge gained to addressing or solving a health problem [12].” mHealth literacy is generally defined as the ability to use health-related apps on a mobile phone [13] or the ability to use mobile devices to search, find, understand, appraise, and apply health information to address a health problem [14]. Although scales for measuring eHealth literacy (eHEALS 1.0 [12] and 2.0 [15]) have been developed, there is no appropriate scale for assessing the unique features of mHealth literacy.

It is of both theoretical and practical importance to develop a scale for measuring mHealth literacy. Theoretically, the notion of health literacy continuously changes in accordance with emerging information technologies. Conventional health literacy concentrates on the abilities to comprehend, evaluate, and apply health information. However, when assessing eHealth literacy, the skills to acquire health information (eg, computer operation skills) and interact with others for support (eg, social interaction) via digital devices are also considered to be crucial components. Likewise, the skill sets for approaching and acquiring health-related resources via mobile phone are becoming more complex. For instance, eHealth literacy scales mainly examine ability to comprehend and evaluate health information, whereas mHealth literacy encompasses not only health information but also important digital resources (eg, health apps, real-time web-based doctor consulting) for health management and improvement [13]. Thus, the usability and multimodality of mobile media allow individuals to conveniently access to various health-related resources. The resources and required skills for mHealth are distinct; yet, they have not been comprehensively represented in existing scales.

Practically, eHealth literacy scales examine the abilities to acquire health information via desktops or laptops, which favors educated and affluent populations; eHealth may not be available to some people, especially those with limited access to computers. In comparison, the convenience and accessibility of mobile devices have led these devices to become pervasive globally; in turn, this has lowered barriers to the use of web-based resources. For instance, more than half of mobile phone users (59.6%) in China are not educated above the junior high level [2]. Therefore, it is essential to design an mHealth literacy scale for diverse population groups, in order to characterize their abilities to access digital health resources.

Previous studies [14,16] have measured mHealth literacy by altering certain phrases in existing eHealth literacy scales (eg, changing “on the internet” to “on a mobile phone”), but these simple word-level modifications do not adequately capture the distinctiveness of mHealth. As reflected in the transition from conventional health literacy scales to eHealth literacy scales, each new emerging health literacy scale is built on the established scales but also shows unique characteristics. Although the concept of mHealth literacy is an extension of eHealth literacy and the domains of these two concepts overlap to some extent, the assessment of mHealth literacy is fundamentally different from that of eHealth literacy. Therefore, mHealth literacy assessment tools need to account for the distinct features of mHealth.

### Objective

We aimed to identify the fundamental components of mHealth literacy for ordinary users and develop a systematic self-report scale that can be used to appropriately measure individuals perceptions of their own mHealth literacy through a problem-based framework.

### Review of eHealth Scales

To operationalize mHealth literacy, it is essential to first understand how health literacy, especially eHealth literacy, is measured in the literature. Conventional health literacy scales (eg, The Test of Functional Health Literacy in Adults [17]) focus on individuals’ abilities to read and comprehend health information as well as their ability to accurately express health problems. eHealth literacy scales emphasize individuals’ abilities to access digital resources to improve health conditions.

There are 3 widely used and empirically validated instruments for assessing eHealth literacy—2 versions of the Electronic Health Literacy Scale, and the Digital Health Literacy Instrument. eHEALS has been widely adopted since the era of Web 1.0 [18,19]. eHEALS comprises 8 self-reported items that measure an individual’s ability to acquire, appraise, and apply health information [18]. However, this scale ignores the fact that people can generate health-related information and interact with each other on the internet. Accordingly, eHEALS 2.0, which incorporates a specific dimension to measure social media interactions, can assess health literacy in the era of Web 2.0 [15]. Although eHEALS 1.0 and 2.0 are valid and reliable instruments, they examine individuals’ abilities to acquire, appraise, produce, and apply health-related resources at a relatively general level—each specific domain (ie, acquiring, appraising, producing, and applying web-based health resources) is measured by a single item. The Digital Health Literacy Instrument [16] addresses this problem by measuring 7 domains of health literacy using 21 self-reported items—each domain is measured by at least three items.

In addition, the Patient Readiness to Engage in Health Internet Technology provides a useful conceptual framework for measuring patient literacy in processing web-based health information. This framework [20] includes eight domains: (1) health information needs; (2) computer or internet experience; (3) computer anxiety; (4) the preferred mode of interaction; (5) the relationship with the doctor; (6) mobile phone expertise; (7) internet privacy; and (8) “no news is good news.” Despite not being validated with empirical data, this conceptual framework still provides insight into the components embedded in digital health literacy.

Because mHealth is an extension of eHealth to the context of mobile communication, eHealth tools can provide the foundation for developing a new mHealth literacy instrument. Indeed, 5 basic domains can be extracted from existing eHealth literacy scales. These include the ability to use digital devices, and the abilities to acquire, comprehend, appraise, and apply health resources with digital devices. In addition, 2 domains proposed under the Patient Readiness to Engage in Health Internet Technology framework—the need for health information and computer anxiety (and internet privacy)—are informative for mHealth literacy because they reflect the motivations and barriers to using electronic and digital health resources. These 7 domains can serve as the foundation upon which the indicators of mHealth literacy are developed.

### The Problem-Based Approach

Most eHealth literacy scales use self-reported items. That is, these scales focus on individuals’ subjective perceptions of and experiences using digital health resources, given that it is difficult to comprehensively measure the operational skills of digital devices (be it a computer or a mobile phone) through an objective test. Nonetheless, such skills are crucial components in eHealth literacy. Self-reported measurement has a number of advantages, namely, easy interpretability, richness of information, motivation to report, causal force, and sheer practicality [21,22] and is thus applicable and useful. Self-perceptions strongly influence the ways in which individuals interact with the world, and subjective experience form the basis of health literacy enhancement interventions [23,24]. In line with previous research [12,14-16,18,19], in this study, we considered the concept of mHealth literacy to be individuals’ perceived abilities in utilizing and managing mobile health resources.

A problem-based approach is particularly suitable for exploring the distinctive features of mobile health resources and clarifying the structure of mHealth literacy. The problem-based framework emphasizes a person’s subjective experiences in solving specific issues. The problem-based framework is modeled after the problem-based learning framework used in medical education, which was initially introduced as a student-centered pedagogy, in which students spontaneously and autonomously learned by solving specific problems [25]. By analyzing a given problem, students were able to determine which skills and attributes they required, and further developed lifelong learning abilities [26].

## Methods

### Overview

Given that an mHealth literacy instrument should evaluate an individual’s ability to solve daily health-related issues, it is reasonable to adopt the problem-based approach as the underlying framework for such an instrument. This study comprehensively examines individuals’ subjective experiences of daily health care practices, including their perception and comprehension of health problems, and their abilities to solve such problems. Specifically, drawing from the problem-based framework, we employ observations and in-depth interviews to explore the specific situations in which mobile users will exploit mHealth resources, as well as to understand the distinct behavioral trajectories of mHealth. Subsequently, we integrate the exploratory findings and the indicators extracted from existing scales to develop the specific indicators.

We conducted in-depth interviews and also observed users to determine their daily mHealth practices, and to identify the specific domains. Then, we assembled a pool of candidate items, based on the domains that we identified, and pretested the candidate items and scale with a small sample. We used item analysis and exploratory factor analysis to identify the most informative items for each domain. Finally, we used confirmatory factor analysis and structural equation modeling to validate and refine the scale that we developed in 2 surveys with large sample sizes.

### Ethics

All empirical studies, as part of a large research project, have been approved by the South China University of Technology (IRB00013151). Participants were informed both the purpose and the process in advance. They were voluntarily involved in the project and could withdraw at any time.

### Participants

All mobile phone users older than 18 years were eligible to participate.

### Exploratory Study to Identify the Domains of mHealth Literacy

In January 2020 in Guangzhou, China, we invited 15 users to investigate how they used their mobile phones to address health problems. Each participant was randomly assigned 1 of 3 types of hypothetical health problems. Participants were asked to read the instructions and then solve the problems with their mobile phones (Multimedia Appendix 1). During the problem-solving process, we observed and recorded participants’ behavioral trajectories of mobile phone use and conducted in-depth interviews to understand their cognitive processes when performing specific actions.

**Figure 1:**
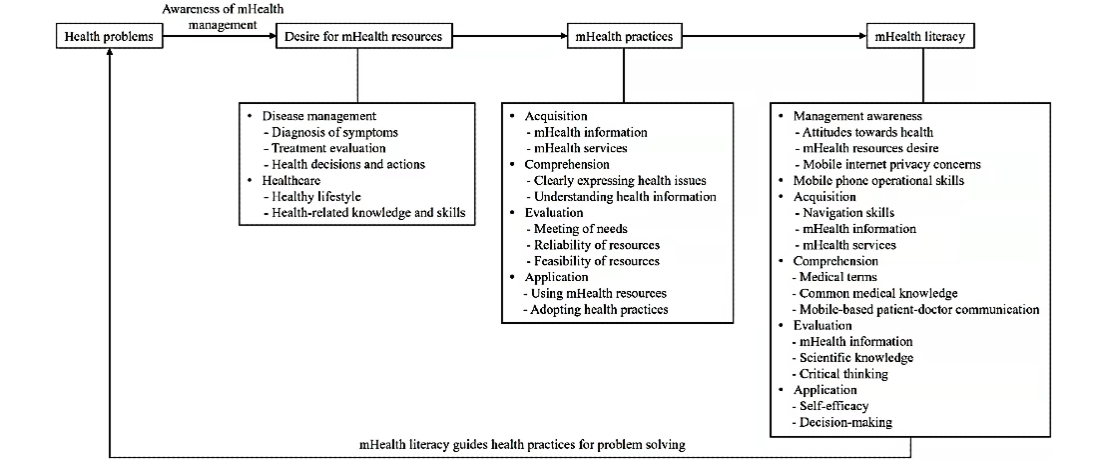
Problem-based framework for mHealth practices and literacy.

We initially identified six main types of mHealth literacy skills (Figure 1): (1) the awareness of one’s health management (including one’s health attitudes, desire for mHealth, and concerns about internet security); (2) operational skills for individuals; (3) the ability to acquire mHealth resources (including navigation skills, information searching skills, and searching for mHealth services via mobile devices); (4) the ability to understand medical information contained in mHealth resources (including the knowledge of medical terms, common medical knowledge, and mobile-based patient–doctor communication skills); (5) the ability to evaluate mHealth resources (including the ability to assess the credibility of mHealth information, as well as the levels of the scientific knowledge and critical thinking skills); and (6) the ability to apply mHealth resources (including self-efficacy, and the ability to make decisions based on mHealth). Since each main type of health literacy skill contained a few key skills, we distributed them across 15 domains in a problem-based framework to guide the construction of our mHealth literacy instrument.

### Development of the Scale

We assembled an item pool by modifying items from existing eHealth literacy scales and creating new items based on the domains identified in the exploratory study. We developed a minimum of 5 items for each domain to ensure that sufficient candidate items were selected. We invited 25 mobile users to test the readability of these items—to check whether their descriptions were comprehensible and consistent with common practices. Based on their feedback, we modified, merged, and deleted various items. The final pool contained 78 items; we assigned a 5-point Likert scale to each of the 78 items.

We then conducted a pilot survey to analyze the psychometrics of the items, determine the reliability of the subscales, and select the most discriminatory items. After recoding reverse items, we performed item discrimination analysis, corrected item-total correlations, and exploratory factor analysis to identify the items that best discriminated the domains and subscales.

We arranged participants’ scores in ascending order and defined those who ranked in the top 27% as high scoring, and those who ranked in the lowest 27% as low scoring. The pass rate was defined as the percentage of participants in a group with 4 or 5 as the response on the 5-point Likert scale; therefore, the *discrimination index* = *pass rate for high-scoring participants* − *pass rate for low-scoring participants*. In this study, we excluded items with a discrimination index <0.3.

Corrected item-total correlations between an item and its domain were calculated; we eliminated items with values less than 0.5 [27].

We used exploratory factor analysis to explore the structure of the items. Studies suggest that approximately 100 to 200 participants should be surveyed in order to have sufficient data for conducting an exploratory factor analysis [28,29]. Thus, we conducted a web-based survey of 148 adult mobile users in February 2020 in Guangdong province, China. We used quota sampling, based on age and sex, to ensure that the demographic characteristics of the sample were representative of the general population of mobile users in Guangdong. We used a principal component analysis with a varimax rotation to extract the factors.

Notably, we did not rely entirely on statistical criteria to select items and construct domains. This was because the selection of variables should be guided by theory and the findings of previous studies [30]. Therefore, we modified the factors and items considering the theoretical frame and statistical results.

### Validation of the Scale

#### Overview

We conducted 2 web-based surveys with large samples. The survey studies were administrated with the Survey Plus applet and Tencent Questionnaire website. Studies indicate that the ratio of items to participants should be between 5 and 10 when confirmatory factor analysis is performed to validate a scale [31]. As with the pilot survey, we used quota sampling (based on gender and age) in both surveys to ensure that the samples sufficiently captured specific demographic characteristics of general internet users.

#### First Survey

The first survey was administrated in March 2020 in Guangdong province, China. We recruited 552 mobile users through a web-based survey to test the scale. The first survey was used to validate the scale that was identified in the pilot test. We first ran confirmatory factor analysis separately for each of the 10 factors and subscales to examine the internal reliability, construct validity, and discriminant validity of each factor and identify a model that fit the empirical data accurately.

Specifically, we evaluated the confirmatory factor analysis model using the *P* value and other indices, including the *χ*^2^/*df* ratio, which is acceptable if the value is between 2 and 5; the goodness of fit index (GFI), which is acceptable if the value is >0.8; the adjusted GFI, which is acceptable if the value is >0.8; the comparative fit index, which is acceptable if the value is >0.9; the nonnormed fit index, which is acceptable if the value is>0.9; and the root mean square error of approximation (RMSEA), which is acceptable if the value is <0.08, with a value <0.05 being optimal [30]. To test the convergent validity of each factor, we evaluated the composite reliability (acceptable if the value is >0.7), the average variance extracted (acceptable if the value is >0.5) [32], and the factor loading of each item (acceptable if the value is >0.6) [33]. We deleted items that had factor loadings less than 0.6 and items with large residual correlations.

Then we compared models by checking goodness of fit indices and their target coefficients. The target coefficient is an index that examines the appropriateness of using a second-order model to substitute a first-order model. This coefficient ranges from 0 to 1, as it calculates the ratio of the chi-square values of the first-order model to those of the second-order model, with acceptable values above 0.7. The closer the target coefficient is to 1, the less information the second-order model loses, and the more suitable it is to use the second-order model in place of the first-order model [34].

#### Second Survey

The second survey, in October 2021, was used to validate the internal and external reliability and the criterion-related validity of the scale. We recruited valid participants nationwide (in China) to improve the generalizability of the results. We adopted exploratory factor analysis and confirmatory factor analysis to retest the validity of the 8 factors and the whole second-order model.

To verify the appropriateness of applying the model to different groups and populations, we conducted multiple-group analysis, using educational level and age, respectively. In addition, to examine the criterion-related validity, we introduced 2 factors that were likely to be associated with health literacy: mHealth use and health prevention behaviors.

mHealth use was assessed with the multiple-choice question “In the past three months, which of the following mHealth behaviors have you engaged in?” with 11 response options (eg, “searching for health or disease information on a mobile phone” and “using health apps on a mobile phone”). The more options the participants selected, the higher the score of mHealth use they received.

Health prevention behaviors (Cronbach α=0.895) was assessed with a 5-point Likert scale. The 10 items in this scale were adapted from the literature [35], where they were mainly used for assessing preventive and protective behaviors during the COVID-19 pandemic (eg, “washing hands after arriving home” and “covering the mouth and nose with a tissue or sleeves when coughing or sneezing”).

Exploratory factor analysis was conducted using SPSS (version 26.0; IBM Corp). Confirmatory factor analysis and structural equation modeling were conducted using AMOS (version 22.0; IBM Corp).

## Results

### Pilot Study

On the basis of our exploratory study, we assembled a pool of 78 items reflecting 15 domains of mHealth literacy. In the pilot test sample, 52% (77/148) of the participants were men and 48% (71/148) were women, and the majority of participants were between 30 and 60 years old (83/148, 56%). After item discrimination analysis and corrected item-total correlations calculation, 60 items remained. We then performed an exploratory factor analysis to extract factors that could reveal the structure of these items.

The Kaiser-Meyer-Olkin value was 0.893, and the Bartlett test of sphericity was statistically significant (*P*<.001), which indicated that exploratory factor analysis was appropriate. We extracted 12 common factors that had a cumulative variance contribution rate of 77.0%. The domains *self-efficacy* and *health practice* were merged; the other factors remained unchanged. After eliminating cross-loaded items and low factor-loading items, 45 items remained. Exploratory factor analysis was repeated a (Kaiser-Meyer-Olkin 0.898; Bartlett test of sphericity *P*<.001), and 11 common factors were extracted from the remaining 45 items, with a cumulative variance contribution rate of 78.7% (Multimedia Appendix 2). The results also indicated a good internal consistency for each factor (all values of Cronbach’s alpha are between 0.777 and 0.921).

We modified the working scale slightly to reflect theory concerning mHealth. The modifications included (1) placing the item “I can identify relevant health information from search results” under Factor 4 (searching for information on a mobile phone); (2) moving the item “The health information accessed from a mobile phone is reliable” to Factor 10 (critical appraisal of information); (3) combining Factor 3 (mobile phone navigation skills) and Factor 4 (searching for information on a mobile phone) to form a new factor, that is, the acquisition of mHealth information; and (4) the addition of a new item, “I can tell whether the acquired health information can solve my problems” into Factor 6 (understanding of medical terms). In sum, we developed a measurement scale with 46 items under 10 subscales scored with a 5-point Likert scale.

### First Validation Survey

A total of 552 respondents (18 to 24 years: n=70, 12.7%; 25 to 30 years: n=134, 24.3%; 31 to 40 years: n=147, 26.6%; 41 to 50 years: n=148, 26.8%; older than 50 years: n=53, 9.6%) participated in the first validation survey. Of these respondents, 52.2% (n=288) were male and 47.8% (n=264) were women; 42.6% (n=235) of participants possessed a high school education (or a lower educational level), the average family monthly income was between 3000 RMB (1 RMB is equivalent to approximately US $0.16) and 10,000 RMB (49.0%, n=271), and 71.0% (n=391) lived in urban areas.

We first ran confirmatory factor analysis separately for each of the 10 factors. The results suggested that the factors were unacceptable, namely, science knowledge and information critique. Therefore, we deleted the factor *science knowledge*, and integrated the acceptable item (information critique) into the factor *evaluating mobile health information*. After deleting items with low factor loadings and items with large residual correlations, 8 factors were retained. The factor loading of each item was larger than 0.6; composite reliability values were between 0.78 and 0.88, and the average variance extracted value of each factor was greater than 0.5 (Table 1).

The square root of average variance extracted for each factor and the Pearson correlation coefficients between factors revealed good construct validity and discriminant validity among the 8 factors (Table 2).

**Figure 2:**
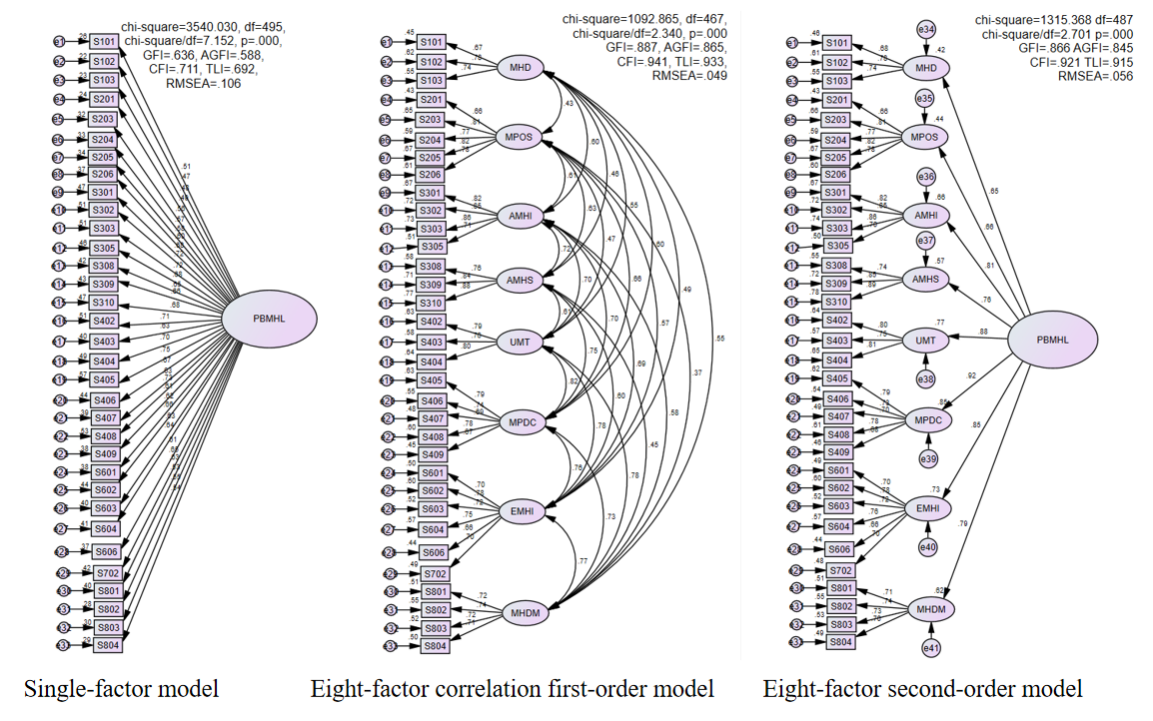
A comparison of the models. Statistic indices — AGFI: adjusted goodness of fit index; CFI: comparative fit index; GFI: goodness of fit index; TLI: tucker-Lewis index. Factor names — AMHI: acquiring mHealth information; AMHS: acquiring mHealth services; EMHI: evaluating mHealth information; MHD: mHealth desire; MHDM: mHealth decision-making; MPDC: mobile-based patient-doctor communication; MPOS: mobile phone operational skills; PBMHL: problem-based mHealth literacy; RMSEA: root mean square error of approximation; UMT: understanding of medical terms.

We compared 3 models to determine which model fit the data best (Figure 2). Model 1 was a single-factor model, in which each item corresponded to an overarching factor. Model 2 was a first-order factor model that included 8 factors that correlated with each other (ie, each item corresponded to a certain factor and subscale, and these 8 factors were correlated with one another). Model 3 was an 8-factor second-order model (ie, each item corresponded to 1 of the 8 factors in the first order, and these 8 factors corresponded to an overarching factor in the second order).

Model 1 could not adequately fit the data; its indices of goodness of model fit fell out of the acceptable range (Table 3); however, the indices for models 2 and 3 were within the acceptable ranges. The target coefficient for both model 2 and model 3 was 0.831 (ie, 1092.865/1315.368). Thus, although both models fit the data well, Model 3 (ie, the second-order model with 8 factors) better reflected the structure of the data. Consequently, we used a second-order model composed of 33 items in 8 domains and subscales to build the Problem-Based mHealth Literacy Scale (PB-mHLS).

**Table 1:** Confirmatory factor analysis results.

Factors and items			Standard Factor Loading		Squared multiple correlations		Composite reliability		Average variance extracted
**Desire for mHealth When encountering health problems that I do not know how to deal with help me.**			—^a^		—		0.78		0.54
	S101: I search the mobile internet for health information.	0.637		0.41		—		—	
	S102: The information found on the mobile internet can	0.821		0.67		—		—	
	S103: I feel that using the mobile internet is a convenient way to solve the problem.	0.731		0.53		—		—	
**Mobile phone operational skills**			—		—		0.88		0.59
	S201: I can operate mobile phones easily.	0.656		0.44		—		—	
	S203: I know how to download new apps.	0.822		0.67		—		—	
	S204: I know how to enter keywords into a search box.	0.764		0.58		—		—	
	S205: I know how to follow officially authenticated accounts on social media.	0.821		0.67		—		—	
	S206: I can successfully purchase goods web-based with my mobile phone.	0.765		0.59		—		—	
**Acquiring mHealth information**			—		—		0.88		0.66
	S301: I know what health resources are available on the mobile internet.	0.825		0.75		—		—	
	S302: I know where to find helpful health resources on the mobile internet.	0.855		0.73		—		—	
	S303: I know how to find mobile-based health resources using my mobile phone.	0.863		0.68		—		—	
	S305: I know how to enter keywords into a search box to find the health resources I need.	0.681		0.46		—		—	
**Acquiring mHealth services**			—		—		0.87		0.68
	S308: I can make a doctor’s appointment using my mobile phone.	0.728		0.53		—		—	
	S309: I know that it is possible to see a doctor for a one-to-one consultation using my mobile phone.	0.848		0.72		—		—	
	S310: I can complete a mobile-based medical consultation with a doctor using my mobile phone.	0.895		0.80		—		—	
**Understanding of medical terms**			—		—		0.83		0.62
	S402: I can understand the explanations given when searching for information about certain symptoms on the mobile internet.	0.757		0.57		—		—	
	S403: I can evaluate the severity of a disease according to the description given on the mobile internet.	0.784		0.62		—		—	
	S404: I can tell whether the health information can solve my problems.	0.823		0.68		—		—	
**Mobile-based patient–doctor communication**			—		—		0.86		0.55
	S405: I can clearly describe my health conditions to an web-based doctor during a mobile phone-based consultation.	0.749		0.56		—		—	
	S406: I can tell the doctor which medicines I am taking during a mobile phone-based consultation.	0.764		0.58		—		—	
	S407: I know that it is possible to take photos of relevant things during a mobile phone-based consultation.	0.727		0.53		—		—	
	S408: I can understand the doctor’s evaluation of my health problems.	0.757		0.57		—		—	
	S409: If I cannot understand the web-based doctor’s explanations, I will tell them so.	0.699		0.49		—		—	
**Evaluating mHealth information**			—		—		0.87		0.52
	S601: I can evaluate the quality of health information available on my mobile phone.	0.692		0.48		—		—	
	S602: I can evaluate the reliability of the evidence cited in mobile-based health information.	0.791		0.63		—		—	
	S603: I usually check the source of health information.	0.732		0.54		—		—	
	S604: I can evaluate the reliability of the source of health information.	0.772		0.6		—		—	
	S606: I search for health information using a variety of channels.	0.645		0.42		—		—	
	S702: I can identify advertisements in search results.	0.671		0.45		—		—	
**mHealth decision-making**			—		—		0.81		0.52
	S801: I am confident in applying the health information that I acquire using my mobile phone to make decisions.	0.658		0.43		—		—	
	S802: I believe that the decisions I make can improve my health.	0.775		0.6		—		—	
	S803: I incorporate my health-related decisions into my daily medical care.	0.746		0.56		—		—	
	S804: I can build a healthy life in accordance with the health-related decisions I make.	0.701		0.49		—		—	

^a^Not applicable or data are not available.

**Table 2:** Discriminant validity test.

	Average variance extracted	mHealth desire	Mobile phone operational skills	Acquiring mHealth information	Acquiring mHealth services	Understanding of medical terms	Mobile-based patient–doctor communication	Evaluating mHealth information	mHealth decision-making
mHealth desire	0.538	0.733^a^	0.427	0.602	0.455	0.546	0.601	0.488	0.547
Mobile phone operational skills	0.590	0.427	0.768^a^	0.615	0.627	0.469	0.657	0.573	0.373
Acquiring mHealth information	0.655	0.602	0.615	0.809^a^	0.721	0.702	0.703	0.685	0.579
Acquiring mHealth services	0.683	0.455	0.627	0.721	0.826^a^	0.608	0.746	0.603	0.453
Understanding of medical terms	0.622	0.546	0.469	0.702	0.608	0.789^a^	0.825	0.781	0.785
Mobile-based patient–doctor communication	0.547	0.601	0.657	0.703	0.746	0.825	0.740^a^	0.762	0.733
Evaluating mHealth information	0.517	0.488	0.573	0.685	0.603	0.781	0.762	0.719^a^	0.771
mHealth decision-making	0.521	0.547	0.373	0.579	0.453	0.785	0.733	0.771	0.722^a^

^a^The square root of the average variance extracted value.

**Table 3:** Model comparisons.

Model comparison	Chi-square (*df*)	Chi-square to *df* ratio	GFI^a^	Adjusted GFI	Comparative fit index	Nonnormed fit index	Root mean square error of approximation
Model 1: Single-factor model	3540.030 (495)	7.152	0.636	0.588	0.711	0.692	0.106
Model 2: 8-factor correlation model	1092.865 (467)	2.34	0.887	0.865	0.941	0.933	0.049
Model 3: 8-factor second-order model	1315.368 (487)	2.701	0.866	0.845	0.921	0.915	0.056
Acceptable values	N/A^b^	<5	>0.8	>0.8	>0.9	>0.9	<0.08

^a^GFI: goodness of fit index.

^b^N/A: not applicable.

### Second Validation Survey

We collected 433 valid and unique responses (men: 222/433, 51.3%; women: 211/433, 48.7%). The majority of respondents were between 30 and 60 years old (263/433, 60.8%); 50.6% (219/433) of participants possessed a middle school education or lower (senior high school: 94/433, 21.7%; college degree: 68/433, 15.7%; a bachelor’s degree or above: 52/433, 12.0%), the average family monthly income was stratified as follows: 3000 RMB and below (112/433, 25.9%), 3001 to 8000 RMB (175/433, 40.4%,), 8000 to 12,000 RMB (93/433, 21.5%), and greater than 12,000 RMB (53/433, 12.2%). The proportion of participants living in urban areas was 50.3% (218/433).

The results of the exploratory factor analysis supported the feasibility of performing factor analysis (Kaiser-Meyer-Olkin 0.955; Bartlett test of sphericity: *P*<.001). As expected, the exploratory factor analysis extracted 8 factors from the 33 items; furthermore, those 8 factors cumulatively explained as much as 81.9% of the variance. The internal reliability of each factor and subscale was also within a good range, as the Cronbach α values for the 8 subscales fell between 0.864 and 0.949 and were greater than the cut-off of 0.7. Overall, these results suggested that the 8 factors extracted through the exploratory factor analysis were appropriate.

Factor loadings of all items were above 0.6, the composite reliability values were between 0.866 and0.947, and the average variance extracted values were greater than0.6. In addition, the second-order model fitness indices were in the good range (*χ*^2^/*df*=2.396, GFI 0.861, adjusted GFI 0.840, comparative fit index 0.949, nonnormed fit index 0.945, RMSEA 0.057).

The result of multiple-group analysis revealed that there were no significant differences in the indices of model fit between separate second-order models for different educational levels (*P*=.31-.47) or different age groups (*P*=.29-.70) (Multimedia Appendix 3). This suggested that the PB-mHLS model was stable across different demographic groups in the sampled population.

The structural equation model to examine the impact of mHealth use on mHealth literacy and the impact of mHealth literacy on health prevention behavior met strict criteria (*χ*^2^/*df*=2.251, GFI 0.860, adjusted GFI 0.840, comparative fit index 0.949, nonnormed fit index 0.945, RMSEA 0.054). The impact factor of mHealth use on mHealth literacy was 0.43, while that for mHealth literacy on health prevention behavior was 0.23. All indicators met the satisfactory significance level (*P*<.001).

## Discussion

### Principal Findings

Using a series of empirical studies—including an exploratory study with in-depth interviews and observations, a pilot survey using a small sample, and 2 web-based surveys using large samples—we developed and validated an mHealth literacy scale that was built on a problem-based framework. Specifically, we constructed a scale with 33 self-reported items in 8 domains and subscales to operationalize mHealth literacy. The PB-mHLS enhances our understanding of the abilities and skills required to use mobile health resources, and extends the notion of health literacy into the context of mobile communication. In this regard, mHealth literacy scales such as the PB-mHLS are likely to retain similar domains or factors that have been identified in eHealth literacy scales. The key components of eHealth literacy mainly relate to the behaviors involved in operating digital devices, and in acquiring, comprehending, as well applying (ie, making health-related decisions) digital health resources (Table 4). These components have been incorporated into the framework of the PB-mHLS. Importantly, this newly developed scale includes unique factors that reflect the distinct characteristics of mHealth. The new scale makes several important contributions to the literature on eHealth and mHealth.

First, unlike the eHEALS and the Digital Health Literacy Instrument, the PB-mHLS integrates mHealth desire as an essential aspect of eHealth practices and integrates this into the measurement of mHealth literacy. Conventional eHealth literacy scales start by evaluating individuals’ behaviors of searching for health resources (eg, acquiring health information), whereas the PB-mHLS extends individuals’ behavioral trajectories by assessing their desire to use the mobile internet to solve health-related problems. In other words, in the PB-mHLS, the starting point for examining individuals’ health behavioral trajectories is their motivations to engage with mobile health practice. This distinction is important because a clear and strong motivation or desire for mHealth is likely to guide an individual’s subsequent actions. This finding was noted in our exploratory study, where participants who were unable to appropriately understand their own health needs tended to either complicate or ignore simple health problems, resulting in worse outcomes. Indeed, when participants were at the same educational levels, those with significant health needs were found to solve their health problems more efficiently. Therefore, the ability to properly express mHealth desire should be a critical component of any mHealth literacy scale.

**Table 4:** Domain comparisons between the Problem-Based mHealth Literacy Scale (PB-mHLS) and eHealth literacy scales.

Domains	eHEALS (Electronic Health Literacy Scale) 1.0 and 2.0	Digital Health Literacy Instrument	Problem-based mHealth Literacy Scale
eHealth behaviors and needs	—^a^	Protecting privacy^b^	Mobile health needs
Digital device operational skills	—	Computer operational skills	Mobile phone operational skills
Acquiring health information	Information navigation awareness; information acquisition skills	Information navigation and searching	Acquiring mHealth information and services
Understanding health information	—	—	Understanding of medical terms
Online interactions	Social media interactions	Adding self-generated content	Mobile-based patient–doctor communication
Evaluation of health information	Evaluation and application of information	Evaluation of information	Evaluation of mHealth information
Application of health information	Evaluation and application of information	Application of information	mHealth decision-making

^a^Not included.

^b^This domain was not confirmed in the as its internal consistency was not acceptable (Cronbach α=0.57) and its item-total correlation was mostly nonsignificant.

Second, the PB-mHLS evaluates individuals’ abilities to use digital or mobile health resources in two ways, that is, by acquiring mobile health information, and by acquiring mobile health services. To evaluate how individuals make use of digital health resources, most eHealth literacy scales include a specific domain for the acquisition of digital health information. However, such widespread use of health information to represent health resources may be less relevant where mHealth is concerned, because individuals can access and adopt various types of real-time medical services conveniently via their mobile phones. As such, individuals’ abilities to acquire health information as well as their abilities to acquire mobile health services are incorporated into the PB-mHLS. This should facilitate comprehensive assessments of individuals’ capacities for using mobile health resources.

Third, the PB-mHLS emphasizes the importance of understanding how different mobile health resources are used by individuals. Just as individuals’ abilities for acquiring mHealth resources include two facets, individuals’ abilities for comprehending mHealth resources are separated into two aspects in the PB-mHLS, namely, the ability to understand medical terms, and the ability to engage in mobile-based patient–doctor communications. the ability to understand medical terms is considered to be an important domain in mHealth literacy because such an ability is salient for reading and understanding the acquired mHealth content, and for accessing and using common medical services such as diagnoses or medical treatments; however, mHealth services provide great opportunities for patients to consult doctors web-based in the absence of any restrictions on time and location. Hence, individual skill in patient–doctor interactions are a crucial factor facilitating mHealth practices, which is included as an important domain in the PB-mHLS.

Finally, the PB-mHLS is an instrument that is focused on assessing individuals’ health-related abilities. While the literature suggests that scientific knowledge should be considered to be a component of eHealth literacy [18], the results of the confirmatory factor analysis in this study did not support such a proposition. Instead of an individual’s general science literacy, an individual’s ability to understand medical terms is a more specific and appropriate factor that can reflect their capacity to comprehend sophisticated medical and health-related information. Similarly, although both the eHEALS and the Digital Health Literacy Instrument included a domain that measures an individual’s ability to engage in web-based or social media interactions, our findings emphasize that web-based interactions represent a focused domain; that is, the interaction between patients and doctors via mobile devices is a more relevant factor in the evaluation of mobile health literacy. In addition, the results suggested that web-based privacy is not necessarily related to mHealth literacy. This finding is consistent with those from previous research [16] and can be explained by the notion of “privacy calculus [36,37].” Although individuals are concerned about web-based and mobile privacy risks, they are willing to trade their private information for health benefits. In other words, their desire to manage and improve their health may outweigh their concerns about privacy risks. As a result, perceptions of privacy risk may influence neither health behaviors nor health literacy, and privacy web-based may not be a necessary component of either eHealth literacy or mHealth literacy.

### Limitations

First, the PB-mHLS should be verified in different contexts. Although the proportion of netizens with a low-education level was accounted for in both of our survey-based studies, the proportion of netizens in rural areas remained unbalanced. Hence, to generalize the PB-mHLS, future studies should apply this scale to populations that are not skilled at using mobile phones, such as people living in rural areas, migrant workers, or low-income groups. Likewise, the development of the PB-mHLS was based on empirical data from individuals in China. Since cultural factors may influence health literacy, it is essential to validate this scale in various other cultural contexts. Second, as was the case in previous studies [16,18,19], we opted to use self-reported questionnaires in developing the PB-mHLS. This method mostly captures self-perceived or subjective responses, and thus, it cannot objectively quantify individuals’ actual abilities. Therefore, future studies should design an instrument that can objectively reflect actual mHealth literacy, and investigate the similarities and differences between actual and self-reported measurements. Third, although the relationships between mHealth literacy, mHealth use, and health prevention behaviors were verified in our study, future research should use the PB-mHLS to examine the relationships between mHealth literacy and other factors, such as health outcomes, actual health practices, or overall health condition, to further validate the appropriateness of this new instrument.

## Appendix

Multimedia Appendix 1 Details of the exploratory study.

Multimedia Appendix 2 Pilot test exploratory factor analysis.

Multimedia Appendix 3 Detailed results of the second survey study.

## Abbreviations

eHEALS: eHealth Literacy Scale
GFI: goodness of fit index
mHealth: mobile health
PB-mHLS: Problem-based mHealth Literacy Scale
RMB: Renminbi
RMSEA: root mean square error of approximation
