# Supplementary material for: Problem-Based mHealth Literacy Scale (PB-mHLS): Development and Validation
Source: JMIR Mhealth Uhealth. 2022 Apr 8;10(4):e31459. doi: 10.2196/31459 (PMC9034416; doi:10.2196/31459)
Supplement: Multimedia Appendix 1 [file mhealth_v10i4e31459_app1.doc]

**Appendix 1. Details of exploratory study**

In the stage of exploratory study, we recruited 15 mobile phone users to investigate how they use the health resources on their mobile phones to solve health problems. The basic information of the participants is as follows:

| **Gender** | **Age** | **Educational level** | **Health status** | **self-perceived mHealth ability** |
| --- | --- | --- | --- | --- |
| Female | 23 | College and above | Good | Strong |
| Female | 27 | College and above | Good | ordinary |
| Male | 22 | College and above | Good | ordinary |
| Female | 48 | Primary school | Not very good | Poor |
| Male | 51 | High school | Bad | ordinary |
| Male | 18 | High school | Good | ordinary |
| Male | 24 | College and above | Good | ordinary |
| Female | 21 | College and above | Not very good | Strong |
| Male | 35 | High school | Good | Strong |
| Female | 32 | College and above | Good | Strong |
| Female | 42 | Primary school | Good | Poor |
| Female | 58 | Primary school | Bad | Poor |
| Male | 19 | College and above | Good | Poor |
| Male | 37 | College and above | Good | Strong |
| Male | 41 | High school | Not very good | ordinary |

We set up three sets of health problems for participants and asked them to solve these problems. We observed this process, after which, we in-depth interviewed them. The first set of problems is the COVID-19 epidemic problem that most people are concerned about, the second set of problems is more relevant to daily health issues, and the third set of problems is to understand scientific and medical knowledge.

| **Health Problems** | **Specific questions** |
| --- | --- |
| ***First set*** | Q1: Can disposable masks be reused? If possible, what methods can be adopted? If not, why? |
|  | Q2:Can self-made masks also have the effect of preventing the coronavirus? If possible, what are the ways to make a mask? If not, why? |
|  | Q3:What concentration of alcohol can be used for daily disinfection of skin surface? Can you spray it directly on your clothes when you get home? |
| ***Second set*** | Q4:Can't sleepwalkers be awakened?why? |
|  | Q5:Can't the neck of an animal be eaten? |
|  | Q6:Is bitter loofah poisonous? |
| ***Third set*** | Q7:Can the currently used aggressive therapies of "oseltamivir" and "arbidol" effectively treat the coronavirus? |
|  | Q8:What is a "super communicator" ? what are its characteristics? |
