# Supplementary material for: Problem-Based mHealth Literacy Scale (PB-mHLS): Development and Validation
Source: JMIR Mhealth Uhealth. 2022 Apr 8;10(4):e31459. doi: 10.2196/31459 (PMC9034416; doi:10.2196/31459)
Supplement: Multimedia Appendix 2 [file mhealth_v10i4e31459_app2.doc]

**Appendix 2: Pilot test exploratory factor analysis.**

| **Factor/Items** | **Factor loading** | **Variance contribution rate** | **Cronbach’s alpha** |
| --- | --- | --- | --- |
| **Factor 1: mHealth desire**  When encountering health issues that I do not know how to deal with... |  | 5.449% | .815 |
| S101: I search the mobile internet for health information. | .842 |  |  |
| S102: The information found on the mobile internet can help me. | .804 |  |  |
| S103：I feel convenient using the mobile internet to solve the problem. | .816 |  |  |
| **Factor 2: Mobile phone operational skills** |  | 10.476% | .912 |
| S201: I can operate mobile phones easily. | .733 |  |  |
| S203: I know how to download new apps. | .778 |  |  |
| S204: I know how to input keywords in a search box. | .845 |  |  |
| S205: I know how to follow official accounts on social media. | .727 |  |  |
| S206: I can successfully purchase goods online with mobile phone. | .799 |  |  |
| **Factor 3: Mobile phone navigation skills** |  | 6.325% | .910 |
| S301: I know what health resources are available on the mobile internet. | .744 |  |  |
| S302: I know where to find helpful health resources on the mobile internet. | .794 |  |  |
| S303: I know how to find mobile-based health resources using my mobile phone. | .769 |  |  |
| **Factor 4: Mobile phone information searching** |  | 3.460% | .792 |
| S304: I know where the search box is on my mobile phone. | .532 |  |  |
| S305: I know how to input keywords in a search box to find the health resources I need. | .683 |  |  |
| **Factor 5: Acquiring mHealth services** |  | 8.015% | .899 |
| S307: I know how to register at a hospital using my mobile phone. | .748 |  |  |
| S308: I can make a doctor’s appointment using my mobile phone. | .811 |  |  |
| S309: I know that it is possible to see a doctor for a one-to-one consultation using my mobile phone. | .804 |  |  |
| S310: I can complete a mobile-based medical consultation with a doctor using my mobile phone. | .755 |  |  |
| **Factor 6: Understanding of medical terms** |  | 6.671% | .854 |
| S403: I know which department I should register with for a specific disease. | .724 |  |  |
| S405: I can understand the explanations given when searching for information about certain symptoms on the mobile internet. | .764 |  |  |
| S406: I can evaluate the severity of a disease according to the description given on the mobile internet. | .666 |  |  |
| **Factor 7: Mobile-based patient–doctor communication** |  | 9.694% | .921 |
| S408: I can clearly describe my health conditions to a online doctor during a mobile phone–based consultation. | .622 |  |  |
| S409: I can tell the doctor which medicines I am taking during a mobile phone–based consultation. | .776 |  |  |
| S410: I know that it is possible to take photos of relevant things during a mobile phone–based consultation. | .681 |  |  |
| S411: I can understand the doctor’s evaluation of my health problems. | .667 |  |  |
| S412: I would tell the online doctor if I could not understand their explanations. | .777 |  |  |
| S413: I would not ask further questions if the online doctor failed to adequately address my questions. | .774 |  |  |
| **Factor 8: Scientific knowledge** |  | 8.028% | .888 |
| S507: A medicine or treatment can be claimed effective only when it has been proven to be effective among most patients. | .635 |  |  |
| S508: It is essential to conduct a series of randomized clinical trials to prove the  effectiveness of a medicine or treatment. | .69 |  |  |
| S509: A medicine or treatment is not reliable if it has not been tested by scientific clinical trials. | .525 |  |  |
| S510: Modern medical knowledge and technologies can improve our health. | .772 |  |  |
| S306: I can identify relevant health information from search results. | .538 |  |  |
| S602: The health information accessible using a mobile phone is reliable. | .54 |  |  |
| **Factor 9: Evaluating mHealth information** |  | 8.024% | .912 |
| S505: I usually check the evidence mentioned in mobile-based health information. | .558 |  |  |
| S506: I can evaluate the reliability of the evidence mentioned in mobile-based health information. | .578 |  |  |
| S512: I usually check the source of health information. | .602 |  |  |
| S513: I can evaluate the reliability of a source of health information. | .730 |  |  |
| S514: I can evaluate the quality of health information available on the mobile phone. | .678 |  |  |
| S515: I search for health information using a variety of channels. | .679 |  |  |
| **Factor 10: Critical appraisal of information** |  | 5.616% | .777 |
| S603: I can identify advertisements in search results. | .677 |  |  |
| S604: I do not click the search results identified as advertising. | .705 |  |  |
| S608: Some of the comments left by users on health websites are not true. | .670 |  |  |
| **Factor 11: mHealth decision-making** |  | 6.951% | .918 |
| S703: I am confident in applying the health information I access using mobile phone to make decisions. | .626 |  |  |
| S704: I believe that the decisions I make can improve my health. | .756 |  |  |
| S705: I incorporate my health-related decisions into my daily medical care. | .738 |  |  |
| S707: I can build a healthy life in accordance with the health-related decision I make. | .692 |  |  |
