# Supplementary material for: Problem-Based mHealth Literacy Scale (PB-mHLS): Development and Validation
Source: JMIR Mhealth Uhealth. 2022 Apr 8;10(4):e31459. doi: 10.2196/31459 (PMC9034416; doi:10.2196/31459)
Supplement: Multimedia Appendix 3 [file mhealth_v10i4e31459_app3.doc]

**Appendix 3. Detailed results of the second survey study**

In second survey, we conducted CFA to re-verify the validity and reliability of this scale. The results of CFA are shown as below:

Table. Results of CFA in the second survey

| **Constructs/Items** | **Indices of Model Convergence** | | | |
| --- | --- | --- | --- | --- |
|  |  |  |  |  |
|  | **Std. Factor Loading** | **SMC** | **CR** | **AVE** |
|  |  |  |  |  |
|  |  |  |  |  |
| **Factor 1 mHealth desire (MHD)**  When encountering health problems that I do not know how to deal with, |  |  | .87 | .70 |
| S101: I search the mobile internet for health information. | .757 | .57 |  |  |
| S102: The information found on the mobile internet can help me. | .876 | .77 |  |  |
| S103: I feel convenient using the mobile internet to solve the problem. | .869 | .75 |  |  |
| **Factor 2 Mobile phone operational skills (MPOS)** |  |  | .92 | .71 |
| S201: I can operate mobile phones easily. | .818 | .67 |  |  |
| S202: I know how to download new apps. | .874 | .76 |  |  |
| S203: I know how to input keywords in a search box. | .882 | .78 |  |  |
| S204: I know how to follow official accounts on social media. | .869 | .75 |  |  |
| S205: I can successfully purchase goods online with mobile phone. | .771 | .59 |  |  |
| Factor 3 Acquiring mHealth information (AMHI) |  |  | .94 | .80 |
| S301: I know what health resources are available on the mobile internet. | .917 | .84 |  |  |
| S302: I know where to find helpful health resources on the mobile internet. | .930 | .86 |  |  |
| S303: I know how to find mobile-based health resources using my mobile phone. | .951 | .90 |  |  |
| S304: I know how to input keywords in a search box to find the health resources I need. | .786 | .62 |  |  |
| **Factor 4 Acquiring mHealth services (AMHS)** |  |  | .87 | .69 |
| S401: I can make a doctor’s appointment using my mobile phone. | .877 | .77 |  |  |
| S402: I know that it is possible to see a doctor for a one-to-one consultation using my mobile phone. | .898 | .81 |  |  |
| S403: I can complete a mobile-based medical consultation with a doctor using my mobile phone. | .912 | .83 |  |  |
| **Factor 5 Understanding of medical terms (UMT)** |  |  | .92 | .80 |
| S501: I can understand the explanations given when searching for information about certain symptoms on the mobile internet. | .877 | .77 |  |  |
| S502: I can evaluate the severity of a disease according to the description given on the mobile internet. | .898 | .81 |  |  |
| S503: I can evaluate if the acquired health information can solve my problems. | .912 | .83 |  |  |
| **Factor 6 Mobile-based patient–doctor communication (MPDC)** |  |  | .95 | .78 |
| S601: I can clearly describe my health conditions to a online doctor during a mobile phone–based consultation. | .863 | .75 |  |  |
| S602: I can tell the doctor which medicines I am taking during a mobile phone–based consultation. | .891 | .79 |  |  |
| S603: I know that it is possible to take photos of relevant things during a mobile phone–based consultation. | .88 | .77 |  |  |
| S604: I can understand the doctor’s evaluation of my health problems. | .907 | .82 |  |  |
| S605: I would tell the online doctor if I could not understand their explanations. | .874 | .76 |  |  |
| **Factor 7 Evaluating mHealth information (EMHI)** |  |  | .93 | .70 |
| S701: I can evaluate the quality of health information available on the mobile phone. | .818 | .669 |  |  |
| S702: I can evaluate the reliability of the evidence cited in mobile-based health information. | .892 | .796 |  |  |
| S703: I usually check the source of health information. | .851 | .724 |  |  |
| S704: I can evaluate the reliability of the source of health information. | .852 | .726 |  |  |
| S705: I search for health information using a variety of channels. | .867 | .752 |  |  |
| S706: I can identify advertisements in search results. | .733 | .537 |  |  |
| **Factor 8 mHealth decision making (MHDM)** |  |  | .94 | .78 |
| S801: I am confident in applying the health information I access using my mobile phone to make decisions. | .874 | .764 |  |  |
| S802: I believe that the decisions I make can improve my health. | .929 | .863 |  |  |
| S803: I incorporate my health-related decisions into my daily medical care. | .891 | .794 |  |  |
| S804: I can build a healthy life in accordance with the health-related decision I make. | .845 | .714 |  |  |

We retest the validity of the second-order model of PB-mHL, the result is shown as below:


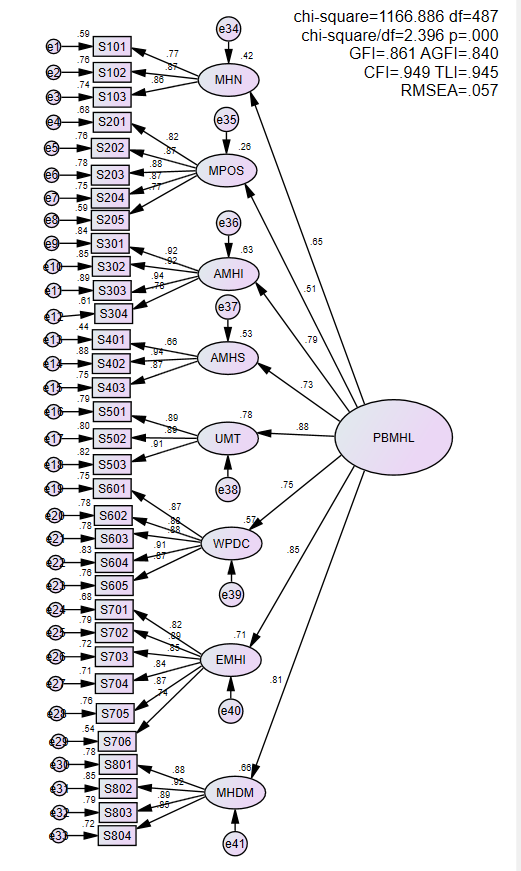


Figure. Results of the second-order CFA model in the second survey

To examine the appropriateness of CFA models across different groups and populations, we conducted multiple-group analysis using educational level and age respectively. The results are as shown below:

Table. Model comparison for different educational groups

| **Model** | **DF** | **CMIN** | ***P*** | **NFI** | **IFI** | **RFI** | **TLI** |
| --- | --- | --- | --- | --- | --- | --- | --- |
|  |  |  |  | **Delta-1** | **Delta-2** | **rho-1** | **rho2** |
| Measurement weights | 25 | 24.911 | .467 | .002 | .002 | -.002 | -.002 |
| Structural weights | 32 | 35.536 | .305 | .002 | .003 | -.002 | -.002 |
| Structural covariances | 33 | 39.244 | .21 | .003 | .003 | -.002 | -.002 |
| Structural residuals | 41 | 50.521 | .146 | .003 | .004 | -.002 | -.002 |

Table. Model comparison for different age groups

| **Model** | **DF** | **CMIN** | ***P*** | **NFI** | **IFI** | **RFI** | **TLI** |
| --- | --- | --- | --- | --- | --- | --- | --- |
|  |  |  |  | **Delta-1** | **Delta-2** | **rho-1** | **rho2** |
| Measurement weights | 25 | 20.829 | .702 | .001 | .002 | -.002 | -.002 |
| Structural weights | 32 | 35.929 | .289 | .002 | .003 | -.002 | -.002 |
| Structural covariances | 33 | 50.527 | .026 | .003 | .004 | -.001 | -.001 |
| Structural residuals | 41 | 70.009 | .003 | .005 | .005 | -.001 | -.001 |

Finally, we introduced external variables including m-Health use and health prevention behaviors to validate the criterion-related validity of PB-mHLS. The result of SEM model is as shown below:


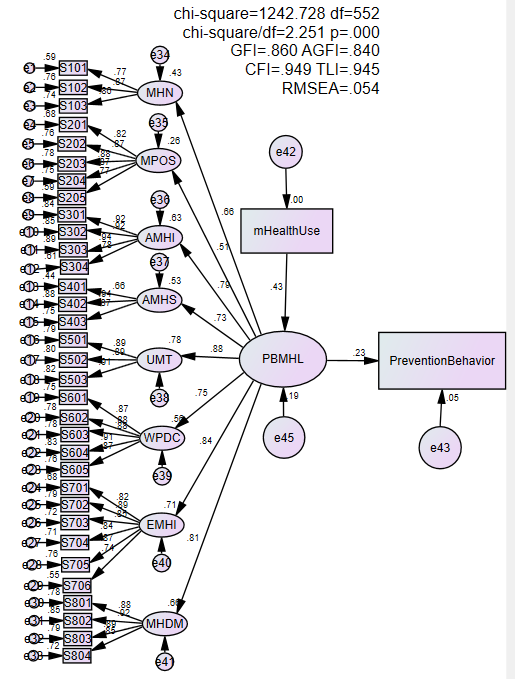


Figure. Results of the SEM model in the second survey
